# A Computational Investigation of Small Peptide of Methyl Jasmonate and Human Complement Factor in Ageing

**DOI:** 10.1155/ijog/9783996

**Published:** 2025-10-30

**Authors:** Oluwafemi G. Oluwole, Ngalla Jillani, Afolake Arowolo, Solomon Umukoro

**Affiliations:** ^1^ Department of Pathology, University of Cape Town, Cape Town, South Africa, uct.ac.za; ^2^ Department of Pharmacology and Therapeutics, Olabisi Onabanjo University, Sagamu, Nigeria, oouagoiwoye.edu.ng; ^3^ Department of Non-Communicable Diseases, Kenyan Institute of Primate Research, Nairobi, Kenya; ^4^ Department of Biomedical Research and Innovation Platform (BRIP), South African Medical Research Council, Cape Town, South Africa, mrc.ac.za; ^5^ Department of Medicine, University of Cape Town, Cape Town, South Africa, uct.ac.za; ^6^ Department of Pharmacology and Therapeutics, University of Ibadan, Ibadan, Nigeria, ui.edu.ng

**Keywords:** human complement factor H, MeJA, MJE1, therapeutic peptide

## Abstract

**Background:**

Ageing contributes to the onset of various diseases. It is accompanied by malfunctioning and deterioration of the body systems. Identifying the biomarkers for the prediction, diagnosis, management, or prognosis of ageing and biological ageing was the aim of this study.

**Methods:**

The peptide identification was done by analysing the conserved sequences in a comprehensive multiple alignment and domain annotation of the small peptide of methyl jasmonate esterase 1 (*MJE1*) present in the *Vitis vinifera* (wine grape). The discovery of biomarkers was done by annotating the RNA‐Seq dataset that comprehensively sequenced the human Achilles tendon transcriptome in young and older people to identify differentially expressed genes. Followed by molecular docking, ADMET and protein–protein interactions studies. The molecular docking was performed by docking the active peptide of *MJE1* with the human complement factor H (*CFH*) identified in the RNA‐Seq data analysis.

**Results:**

The sequenced alignment analysis indicated high similarity (~99%) of *MJE1* protein in the *Vitis vinifera* (wine grape), as well as some other set of plant species that also encode a gene for *MJE1*. The pharmacokinetics and ADMET properties of MJE1 revealed a molecular weight of 224.30 g/mol and polar surface area measured at 43.37 Å^2^, suggesting its adherence to Lipinski’s rules of for drug likeliness; moreover, data supported MJE1 interactions with mostly nuclear receptors. Of all the annotated biomarkers in the RNA‐Seq dataset analysed in this study, we prioritised the *CFH* for the robust data supporting its activities in ageing research and the availability of its crystalline structured. The molecular docking of MJE1 selected peptide with *CFH* surface identified conformational changes enacted by the surface and orientation interactions with four amino acid residues (CYS 1167, THR 1227, TRP 2 and PHE 10) of the protein.

**Conclusion:**

By using computational techniques to investigate the plausible anti‐ageing potential of the small peptide of *MJE1* present in the *Vitis vinifera,* we identified a complex interaction between *MJE1* and *CFH*. This finding necessitates further investigation in mouse, zebrafish or non‐human primate model towards the understanding of anti‐ageing therapy.

## 1. Introduction

The number of ageing population is rapidly increasing. The ageing population is projected to grow to 1.4 billion by 2030 and 2.1 billion by 2050 [1]. An ageing population presents many challenges in employment, learning, integrated health and long‐term care systems.

The ageing process starts early in life for many species and becomes more pronounced in more complex organisms. It has been suggested that early degenerative changes in tendons result from an accumulation of micro‐damage within the extra‐cellular matrix (ECM) due to an imbalance between anabolic and catabolic pathways. The ageing process affects the whole body, but its manifestations can differ depending on the individual and on what tissues and organs are being affected. Likewise, biological ageing is the formation of molecular and cellular damage that causes decline in function and increased risk of disease. Of note, the mechanisms of ageing are not fully understood to date. There is a clear need to study the biomarkers for prediction, diagnosis, management and prognosis in ageing. Little is known about the role of immune‐mediated biomarkers such as rheumatoid factors and stress‐mediated biomarkers such as cortisol in ageing. In addition, there is increasing evidence to support the roles of neuroendocrine biomarkers in organ‐specific ageing [2]. It is not fully understood how these biomarkers affect the ageing processes in diverse populations. Studies in these aspects can illuminate the ageing processes and the significance of biomarkers in ageing to precision medicine. Many factors that contribute to ageing have been highlighted in literature [3, 4]. However, little evidence exists to underpin novel biomarkers that can be targeted by novel therapies to reduce the impacts of biological ageing. Ageing is generally associated with a decline in protein synthesis, and intake and a loss of cell functionality [5, 6]. The ageing processes primarily affect the muscles, bones and joints. It has been suggested that early degenerative changes in tendons are a significant observation in ageing [7, 8]. The process of ageing can be influenced by factors like genetics and lifestyle (diet, exercise, stress). Cell ageing has been associated with a decreased ability to modulate inflammation. Also, tendon degeneration can be associated with various age‐related movement disorders and neurodegenerative diseases like Parkinson’s disease, ataxia and muscle dystrophy [9]. While ageing could be genetic, some minor genetic variations in DNA can also correlate with phenotypic changes and how individuals respond to medication or the risk of getting a disease. Often, these variations occur within the DNA letters that make up the protein‐coding portion of a gene, influencing how the protein works. The gene expression approach has been used to quantify the increasing or decreasing amount of RNA or protein made from a particular gene, which can influence biological processes in normal or disease states [10].

Plants are known to utilise their innate immune system to protect themselves against hazardous situation. Hence, they are constantly being investigated for mechanisms and therapeutic potentials. For example, an integrated network pharmacology, novel peptidomics, bioinformatics screening, and biochemical and genetic validation methods for peptide identification can improve drug design. In addition to many non‐protein regulators, various phytohormones play distinct roles in plant defence responses. Plants or compounds rich in salicylic acid, jasmonic acid, ethylene and ascorbic acid are all traditional plant hormone signalling molecules. Salicylic acid and jasmonic acid/ethylene also exert antagonistic effects on other plant physiological processes, including root growth, stomatal regulation, photosynthesis and flower development. Salicylic acid‐based drugs are increasingly common; however, most jasmonic acid drugs are currently in clinical trials. The process of getting jasmonic acid/jasmonate mainly depends on the hydrolysis of methyl jasmonate (MeJA) to jasmonate. Hence, peptides associated with this process must be identified and annotated to achieve more accurate and precise therapeutic effects.

*Vitis vinifera* (wine grape) is a species of *Vitis* belonging to the Vitaceae family. It is most native to the Mediterranean region, central Europe, Southwestern Asia, Morocco, Portugal, Southern Germany and northern Iran. It is a woody vine that is widely used to make wine and raisins. Few studies have shown that wine grapes have medicinal properties, and the DNA sequences of wine grapes encode several conventional and non‐convectional peptides. Still, very few studies have investigated their functions [11]. One of the peptides of *V. vinifera* that is not yet thoroughly investigated is methyl jasmonate esterase 1 (*MJE1*). *MJE1* is known to help plants produce MeJA and jasmonate to prevent damage. Hence, the peptide can be targeted for production and modifications to act on some biomarkers of diseases directly. Peptide drug development has significantly progressed in the last decade due to the ability to modify naturally occurring plant peptides [12]. Plant peptides play integral role in the modulations of various biological pathways due to their diverse functions. The usefulness of crude plant extracts as an alternative therapy to treat ailments is due to their affordability and widespread availability [13]. Natural products from plants have been the source of valuable drugs such as aspirin, reserpine, physostigmine and quinine [13]. By exploring the potential of natural peptides through a reverse pharmacology approach while prioritizing safety profiles may represent a rational strategy in precision medicine. Precision medicine has the potential to transform how diseases are prevented, diagnosed and treated, and it is already impacting some areas of medicine [14–16].

The practical applications of the knowledge of three‐dimensional structures of ligand–target complexes emerged in the last few decades. The possibility of solving the three‐dimensional structure of the active site became increasingly available. Considering the importance of the application of computational methods, as an allied technology in the evaluation and validation, of the dry response hypothesis, in molecular design and after a brief description of active sites, and enzyme structure, this work will concentrate on the universal method of molecular docking in drug discovery process available to all theoretical chemists working in the discovery of new therapeutic agents. By using multiple approaches to understand molecular docking and predicts the conformation and orientation of ligands in the active site, researchers can be able to narrow down to a particular target and facilitate the development of effective targeted therapies. The data that we used in this study were derived from a repository that catalogued RNA‐seq expression obtained from the tendons from young and old donors. The present study aimed to identify differentially expressed tendon transcripts in ageing to characterise molecular mechanisms associated with age‐related changes in tendons. And to perform computational analyses to investigate the small peptide sequences derived from *Vitis vinifera* for plausible therapeutic interactions with ageing‐related biomarkers.

## 2. Methods

### 2.1. Peptide Characterisation and Selection

From the sequenced genome of the French–Italian Public Consortium for Grapevine Genome Characterization 2007, the wine grape (*Vitis vinifera*) sequences were retrieved and a BLAST run was conducted on NCBI. The proteins of wine grapes (*Vitis vinifera*) were accessed on PubChem (https://pubchem.ncbi.nlm.nih.gov/). The protein selected for study is MJE1. This selection was based on the literature review on molecular, biochemical and structural studies that confirmed that MJE1 hydrolyses MeJA into jasmonate. The protein sequences were retrieved and a BLASTP was run on NCBI to obtain a comprehensive multiple alignment of multiple sequences alignment and identify species with MEJ1 peptides and MeJA activities (Figure S1). The ancestral protein sequences of MEJ1 were further analysed for the active domains using the InterPro tool by submitting the sequences to the InterProScan web service for scanning against the InterPro protein signature databases.

### 2.2. Computational Pharmacology and Pharmacokinetic Properties

MJE1 activities produce MeJA activation, a more stable compound. Hence, the canonical SMILES notation of MeJA (CC/C=C\CC1C(CCC1=O)CC(=O)OC) and the purified peptide _1_GEWDNTWNSAZUDX‐PLNGDYQASA‐N_25_ derived from the PubChem were utilised to explore the pharmacokinetic properties and absorption, distribution, metabolism, excretion and toxicity (ADMET) analysis of MeJA in Swiss ADME. The drug‐likeliness of MeJA was investigated according to Lipinski’s rules which address the compound’s oral bio‐availability (OB ≥ 30), molecular weight (MW< 500 Da), drug‐likeness (DL ≥ 0.18), hydrogen bond donors (H donor < 5), octanol‐water coefficient (P < 5) and hydrogen bond acceptors (H acceptor < 10). The target prediction was investigated using the SwissTargetPrediction (http://www.swisstargetprediction.ch/).

### 2.3. Discovery of Ageing Novel Targets Through Gene Expression Data Profiling

The gene expression data were retrieved from the Bgee database for post‐processed analysis. The Bgee database catalogued various gene expression patterns across multiple animal species, and the data can be retrieved for further analysis and comparison. This study downloaded the RNA‐seq data (E‐MTAB‐2449) of Achilles tendon from young and old donors. The experiments were done using samples donated by normal individuals (*Homo sapiens*) categorised as young (ages 14–27) and old (ages 60–79). The assays were done in replicates for each individual (females *n* = 3, males *n* = 6). The experiment aimed to identify differentially expressed tendon transcripts in ageing to characterise molecular mechanisms associated with age‐related changes in tendons [17]. Our study, therefore, utilised the publicly available bulk RNA‐Seq processed expression and FASTQ file from the experiment to discover relevant information to the study objectives. In the current analysis, we prioritised only data for donors of 14, 21, 74 and 79 years. The FASTQ files were processed to validate the quality. The files generated by the processed expression values were downloaded from (https://www.ebi.ac.uk/biostudies/arrayexpress/studies/E-MTAB-2449/sdrf) to the local terminal. The pre‐processing of the FASTQ files and quality control were done with FastQC v 0.12.1, Fastp v.0.23.4, cutadapt 4.4 with Python 3.12.3 and MultiQC v1.18. Reads were aligned to the reference genome (Ensembl build GRCh37) using TopHat. The Deseq2 Galaxy tool collated the list of differentially expressed genes and the data were compared with the bulk processed RNA Seq results retrieved from the Bgee database. The tidy‐verse and dply packages in R were used to pre‐process of the Tab‐separated values (TSV) files. The values were then filtered with stringent criteria using the following approaches:

Every element of enumeration has a different format:

1. The transcript per million (TPM) is greater than 10
2. The *p* value is less than 10^−15^
3. The read count representing the number of reads mapped to the gene is greater than 10^3^.

By using these criteria, we sought to reduce noise and identify abundantly expressed genes. The stringent criteria filtered the high TPM, read count, fold‐change thresholds or statistical significance available to identify differentially expressed genes enriched in older donors. Subsequently, the visualisation and further analyses were done with ggplot in R. The function rcorr() [in the Hmisc package] in R was used to compute the significance level for Pearson and Spearman correlations between variables and factors.

### 2.4. Molecular Docking of MJE1 With Human Complement Factor H (CFH)

The filtering of the gene expression data identified specified genes as potential candidate genes in ageing studies; however, the human complement factor H was selected for the molecular docking from the top‐selected genes. In our study, the ligand for docking is MJE1. But we can only dock a small peptide from it. So, we first identified the most active site through InterPro and sequence alignment with other species. The protein for docking is CFH. We selected this based on the filtering and statistical threshold of differentially expressed genes in old donor samples only. There are more, but we prioritised the CFH because it has an existing crystalised structure in PDB; therefore, its visualisation and exploration were possible with PyMOL. For the molecular docking, we now used the CAB‐Dock as described: Random structures of the peptide are generated in the simulation of binding, and the docking procedure utilises replica exchange Monte Carlo dynamics (50 cycles) with 10 replicas uniformly spread on the temperature scale. On output, the procedure produces 10 trajectories (one for each replica), each consisting of 1000 time‐stamped simulation snapshots for a combined total of 10,000 models. The reconstruction of the 10 final models undergoes optimisation using a Modeller with DOPE statistical potential that reflects the slope of the penalty curve. The protein 3D structures of CFH Carboxyl Terminal Domains 19‐20 (PDB: 2G7I) protein were obtained from the RCSB PDB in PDB format (https://www.rcsb.org/) which represented the best protein crystal structure for docking, emphasising smaller resolution (1.75 Å), completeness and human origin. The FASTA sequence of the most suitable model was downloaded and a BLAST run was performed in NCBI. Protein structure was refined by using the software PyMOL V2.5.5. Following this step, the ligand and protein molecule values, i.e., the structure factor and the validation 2fo‐fc coefficients files were also retrieved for the post‐docking comparison. The long‐chain peptides of MeJA were selected based on the InterPro domain features and fragment length. The small peptide ‘AWCWYKVTTFLRSAGHKVTALD’ was docked with the 3D structure and protein isoform of CFH. The molecular docking with the CABS‐dock tool set at 50 simulation Monte Carlo (MC) cycles.

## 3. Results

### 3.1. Identification of *MJE1* Peptide in Some Other Plant Species

**Figure 1:**
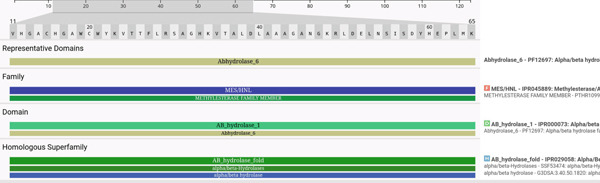
The protein domain annotation track of MES/MeJA with InterProScan. It revealed the functional sequences of proteins by classifying them into families and predicting domains and important sites. The *homologous superfamilies* identified three homologous hydrolase annotation tracks with encoding peptide sequences on the ProtVista view for the structural annotations.

The analyses of the *MJE1* protein sequences obtained from the *Vitis vinifera* genome identified multiple plant species with high similarity scores. For example, the *Theobroma cacao* and *Quercus* family have more aligned contigs. Others include the *Mangifera indica* (Figure S1). The NCBI Multiple Sequence Alignment Viewer, Version 1.25.2, shows a similarity score. The alignment of the positive strand of sequence span from 0 to 699 nucleotides to determine the similarity score. Also, the domain analyses of MJE1 revealed the most active domain is MES‐1, i.e., AB‐hydrolase 1 and 6. The frame fine‐tuning highlights the 54 amino acids encoding the AB‐hydrolase 1 and 6 domains enriched with alpha/beta hydrolase chains. This analysis is consistent with the affirmation of the MES‐1’s role in the hydrolysis of MeJA to jasmonate (Figure 1).

### 3.2. The Identification of Drug‐Likeliness in *MJE1* Activities

**Figure 2:**
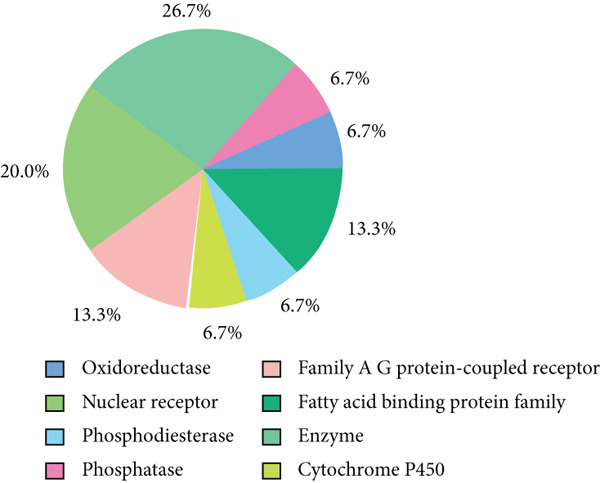
The top 15% predicted biological systems and targets of MeJA. The robust pathways in inflammation, oxidative stress and DNA damage.

The physicochemical properties of the derivative of *MJE1*, i.e., MeJA protein are highly acceptable for drug likeliness and target optimisation. The molecular weight was 224.30 g/mol, and the topological polar surface area value was 43.37 Å^2^. It is highly soluble with a high Log S value of − 2.47. The ADME results showed that it was highly absorbed in the gastrointestinal tract (GI) and the by‐product can cross the blood–brain barrier (BBB). Notably, it was not inhibited by the most common cytochrome enzymes (CYP1A2, CYP2C19, CYP2C9, CYP2D6 and CYP3A4). The peptide fulfilled Lipinski’s drug‐likeness rules by having a bio‐availability score of 0.55 and a synthetic accessibility score of 3.38 (1 [very easy]–10 [very difficult]). The SwissTargetPrediction analysis in the report identified 100 biological targets directly implicated in various mechanisms such as reactive oxygen species, inflammation and stress hormone regulations. The top 15% of biological systems target of interest are plotted in (Figure 2). This data indicates that the peptide has strong activity on nuclear receptors and regulatory enzymes and can be targeted to produce improved therapeutic or pharmacodynamic effects.

### 3.3. Identification of Novel Candidate Genes in Age‐Related Gene Expression Changes

By processing the FASTQ files (R1/R2) used for generating the gene expression data (RNA‐seq data E‐MTAB‐2449), the quality check and trimming of duplicate reads were performed in this study as well. The MultiQC data showed that 95.0% of the called bases have a Phred score of more than 30, 97.6% have a Phred score of more than 20, and a base ratio of 0.2–0.3 (Figure S2). A ‘base‐content ratio plot’ in FastQC, also called the ‘per base sequence content’ plot, essentially shows how the balance of bases changes across the read length, allowing you to identify potential biases or issues related to nucleotide composition in your sequencing data. The Deseq2 analysis identified 332 differentially expressed genes in the combined dataset (*p* < 0.05). The result closely validated the previous results from the first study that generated this data and the first analyses. The complete list of all genes mapped can be assessed on Bgee E‐MTAB‐2449. The stringent filtering criteria used in this study ensure that very relevant genes associated with ageing tendons are evaluated. Figure 3 shows the heat map graphical representation and association of the top genes prioritised for further analyses. The gene expression data distribution across all datasets was represented in the Q‐Q plot where the normal quantiles distribution data values vary slowly over a small range with a few extreme values (Figure S3). A Q‐Q (quantile–quantile) plot is used to see if a dataset follows a particular theoretical distribution. It works by comparing the quantiles of the observed data to the quantiles of this other distribution. The line is constructed by calculating the slope and intercept using the first and third quartiles of both the observed and theoretical distributions. Clearly, the data did not show normal distribution, hence suggesting that the probability of obtaining each value randomly might be very low. The stringent filtering criteria and other modifications of the top selected genes identified a list of genes in Table 1 that were differentially expressed in the tendon from young and old donors. Similarly, there was a correlation between the read counts and the donor’s gene expression level (Figure 4). Nonetheless, the *CFH* gene was prioritised for molecular docking because it is the only gene specifically differentially expressed in the old donors and has also been fully characterised in the PDB as having unique isoforms of human origin.

Figure 3The heat map graphical representation of statistical significance correlated genes based on their expression values and derived *p* values. The data highlight genes with statistical differences (*p* < 0.001) in (a) 14‐year‐old donor, (b) 21‐year‐old donor, (c) 74‐year‐old donor and (d) 79‐year‐old donor.

**Figure:**
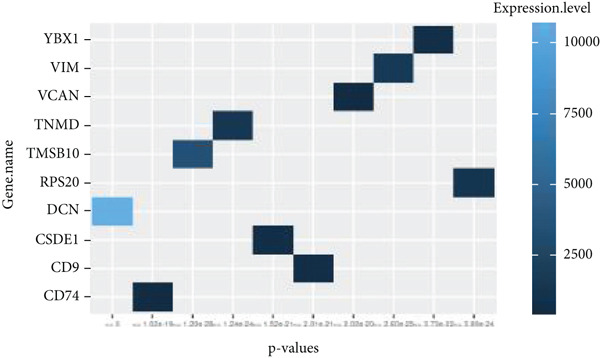
(a)

**Figure:**
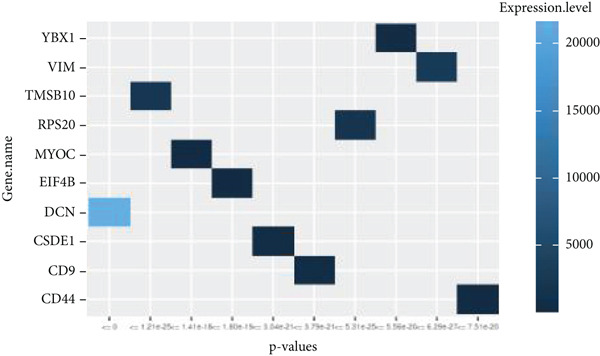
(b)

**Figure:**
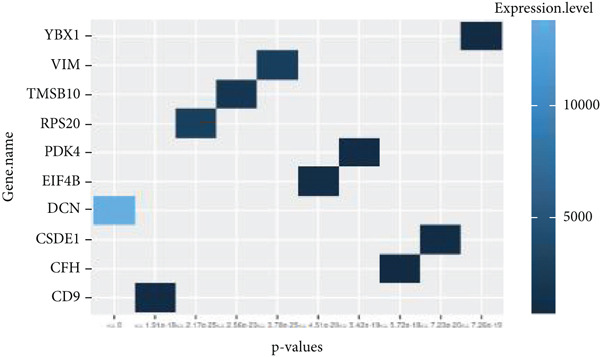
(c)

**Figure:**
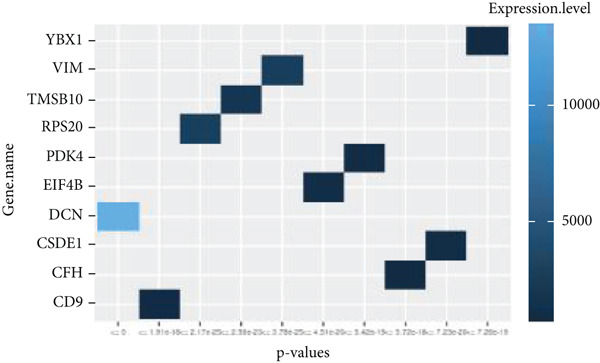
(d)

**Table 1:** The top selected candidate genes.

**Gene symbol**	**Expression level (TPM)**	***p* value**	**Age**
*DCN*	10,684.823692	0	14
*TMSB10*	3436.137219	1.2E−28	14
*VIM*	1641.690218	2.6E−25	14
*TNMD*	1403.999206	1.24E−24	14
*RPS20*	1250.034653	3.89E−24	14
*YBX1*	776.46333	3.73E−22	14
*CSDE1*	667.715537	1.52E−21	14
*CD9*	647.668102	2.01E−21	14
*VCAN*	502.328809	2.02E−20	14
*CD74*	418.345503	1.02E−19	14
*DCN*	22,323.998963	0	21
*VIM*	3247.933522	6.29E−27	21
*TMSB10*	2453.064381	1.21E−25	21
*RPS20*	2125.596728	5.31E−25	21
*CSDE1*	881.468421	3.04E−21	21
*CD9*	861.037586	3.79E−21	21
*YBX1*	643.846675	5.56E−20	21
*CD44*	622.88911	7.51E−20	21
*EIF4B*	565.151243	1.8E−19	21
*MYOC*	448.204333	1.41E−18	21
*DCN*	14,224.87217	0	74
*RPS20*	2696.288346	2.17E−25	74
*VIM*	2549.825164	3.78E−25	74
*TMSB10*	1652.070834	2.56E−23	74
*EIF4B*	730.129468	4.51E−20	74
*CSDE1*	691.877521	7.23E−20	74
*PDK4*	547.913098	5.42E−19	74
*YBX1*	529.36286	7.26E−19	74
*CD9*	472.105624	1.91E−18	74
*CFH*	413.836349	5.72E−18	74
*DCN*	14,491.437963	0	79
*RPS20*	2533.564738	2.19E−25	79
*VIM*	2129.635184	1.23E−24	79
*TMSB10*	1950.076196	2.94E−24	79
*MYOC*	1641.223565	1.57E−23	79
*CSDE1*	835.403772	8.58E−21	79
*YBX1*	600.680268	1.6E−19	79
*CD9*	591.123487	1.84E−19	79
*EIF4B*	522.461903	5.36E−19	79
*PPP1R12A*	343.747707	1.81E−17	79

Figure 4The read count and expression value relationship visualised in a few selected genes. The data suggest the abundance reads have no statistical differences (*p* < 0.01) in terms of gene expression in (a) 14‐year‐old donor, (b) 21‐year‐old donor, (c) 74‐year‐old donor and (d) 79‐year‐old donor.

**Figure:**
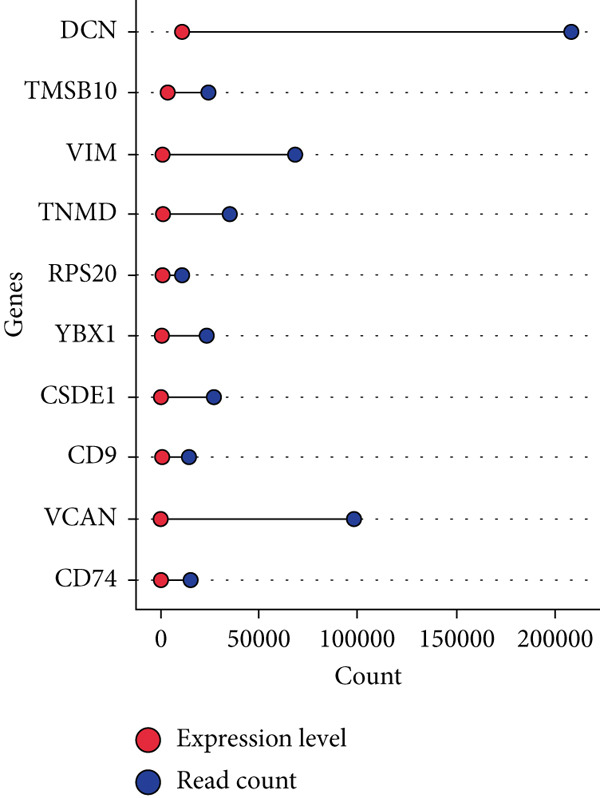
(a)

**Figure:**
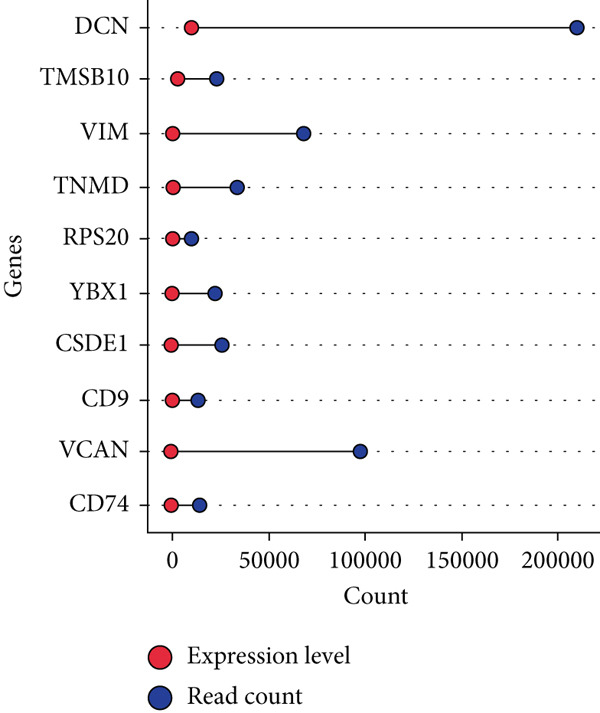
(b)

**Figure:**
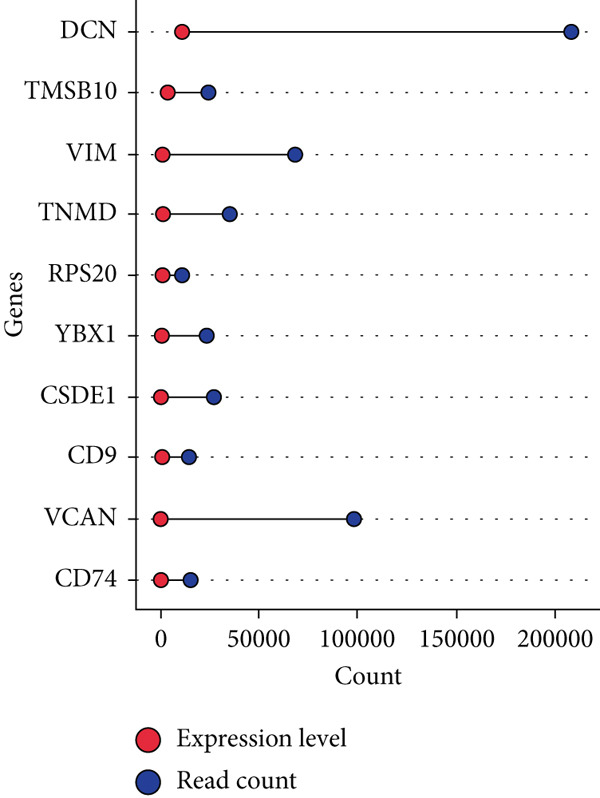
(c)

**Figure:**
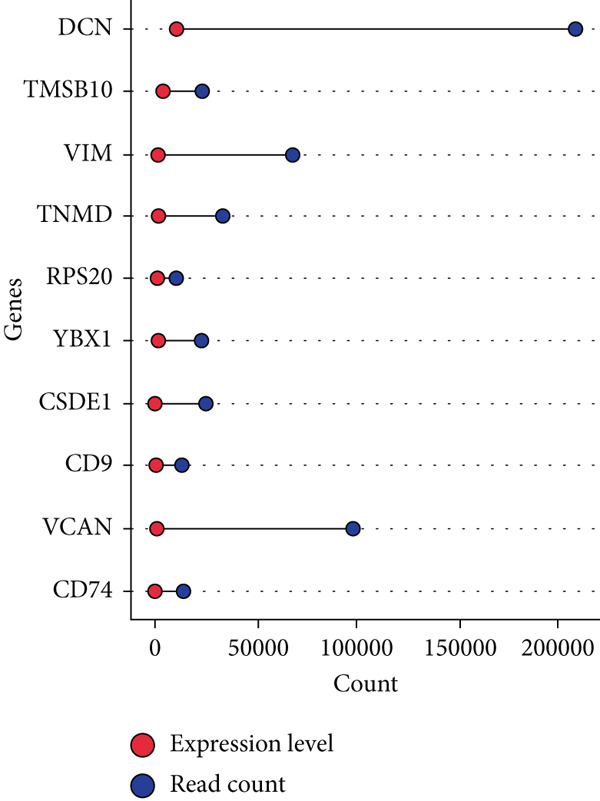
(d)

### 3.4. Therapeutic Potentials Observed in MJE1 and CFH Complex

Docking is a prediction method where peptide residue binds with the target receptor or protein. The *MEJ1* and *CFH* complex analyses to determine the binding sites residues indicated that the protein‐peptide complex has an affinity and the residues play a significant role in efficient binding (Figure 5a). The results displayed 10 models. The top model has 10 trajectory clusters used for the prediction. The model has 217 elements, a cluster density score of 38.9444, and a max RMSD value of 34.1159. The pairs of peptide/receptor residues within 4.5 Å in the selected complex show the residues that dictate the interaction and strong interaction with the target receptor (Figure 5b). The best orientation indicates that the protein interacts with four amino acid residues of the peptide residues, i.e., CYS 1167, THR 1227, TRP 2 and PHE 10. This orientation and interaction led to the bending of the complex (Figure 5c), thereby indicating that it confers biological activity and chemical stability.

Figure 5(a) The docking analyses of protein–peptide complex showing affinity. (b) The interaction map of the residues. (c) Residue with the strongest orientation interaction that could bend the complex and may confer biological activity and chemical stability.

**Figure:**
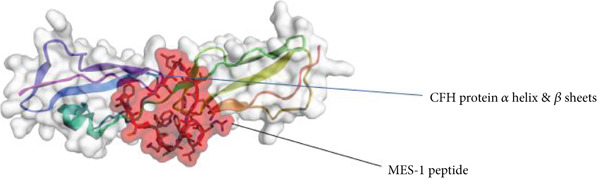
(a)

**Figure:**
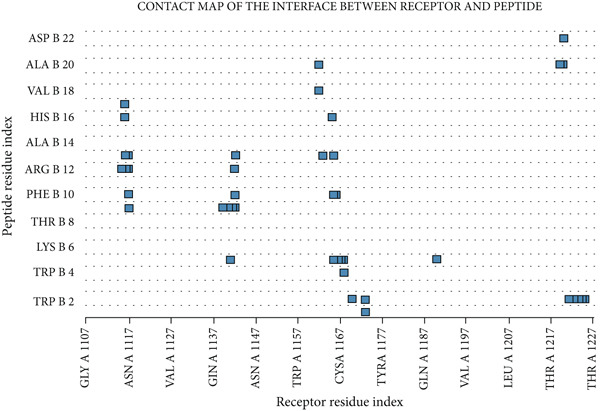
(b)

**Figure:**
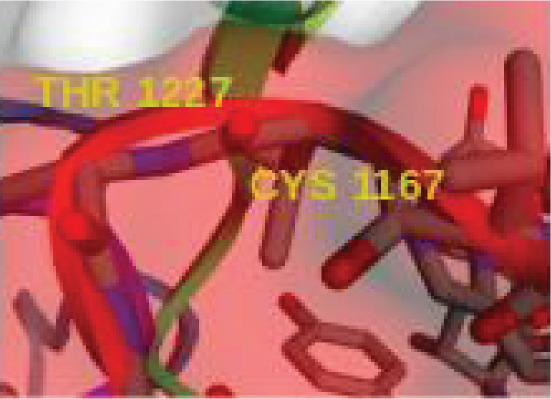
(c)

## 4. Discussion

The findings from our study highlighted the need to investigate plant‐based peptide for the treatment of ageing‐related diseases. Our study revealed the potential mechanisms by which the *MJE1* derived from *Vitis vinifera* (wine grape) could be acting through the binding of various receptors like *CFH*. Various causal factors and biomarkers have been linked to ageing‐related diseases, particularly those that affect muscles and movements, e.g., Parkinson’s disease [18–21]. For example, the MAPK signalling pathway and the eventual generation of reactive oxygen species can trigger immune responses [22, 23], whereby damaging the membrane of cells and enact processes that induce inflammation, which some medicinal plants have been shown to ameliorate in animal models [24, 25]. To target treatments, traditional biochemical approaches have been used to validate the biological functions of plant peptides. For example, we reported on the biochemical activities of MeJA in animal models [26, 27]. The ancestral protein sequence alignment is supported in literature [28]. Various plant species synthesise *MJE1* protein as shown in the ancestral protein sequences alignment in this study. For example, *Mangifera indica* is one of them and has also been showed to produce a strong anti‐inflammatory and immune responses in animal models [29, 30].

The BLAST tool that we used has been recommended by various researchers globally. The tool enabled us to find regions of similarity between sequences. The program compares nucleotide or protein sequences and calculates the statistical significance of matches. The BLAST inferred evolutionary relationships between sequences as well as enabled us to identify members of different species that possess *MJE1*. The output corroborates with the functional identification of the active sites of *MJE1* by the InterPro tool. The InterPro provides functional analysis of proteins by classifying them into families and predicting domains and important sites. We trimmed the sequence to AWCWYKVTTFLRSAGHKVTALD, which encodes the hydrolase enzyme. Hydrolases are the group of enzymes that catalyse bond cleavages by reaction with water. The natural function of most hydrolases is digestive to break down nutrients into smaller units for digestion. Hydrolases are generally extracellular enzymes that can be easily purified.

The molecular docking estimated the binding free energy by first identifying the complementarity of the receptor–ligand binding site, using a fast calculation based on atom–atom distance to take advantage of the geometric complementarity found in rigid protein–ligand docking studies [31]. Molecular docking is considered to be a promising strategy for the discovery of new peptide drug candidates. The discovery of potential therapeutic peptides is the first step of peptide drug development, followed by chemical or biological peptide synthesis and sequence modification to improve its pharmacological properties.

The CABS‐dock allowed us to understand the potential interaction of MJE1 peptide with CFH by performing global and local docking. Global docking ensures the interaction across all residue, and the local docking when a site/interaction has been found, it does additional modelling to predict correct interaction, and as well as it gives up to 10 models to choose from. Each docking simulation generated 10,000 C*α*‐trace models, sampled without prior knowledge of the binding site. The models were scored using CABS internal energy terms, followed by structural clustering to identify the top 10 candidates. It uses replica exchange Monte Carlo sampling to explore peptide–protein interactions. Within the method, the resulting models are reconstructed to all‐atom resolution. The (50) number of Monte Carlo macrocycles is the standard threshold recommended to perform this analysis. This threshold can only be increased if the *MJE1* peptide and CFH have a large surface area for the interaction. The ligand was gradually sampled within the confined geometry by using flexible protein attachment to the van der Waal spheres. The analyses gave us the insights about the plausible association reported in this study.

The CABS‐dock itself does not produce a standard binding affinity score (like a pKi or Kd); instead, it uses the CABS interaction energy to rank the models of protein–peptide complexes generated during the docking simulation. The scoring by the CABS‐dock produces a cluster density score of 38.9444 and a max RMSD value of 34.1159. The pairs of peptide/receptor residues of 4.5 Å. The interpretation of the binding affinity here is the 4.5 Å. A ligand‐RMSD of less than 3 Å denote high‐accuracy of interaction, medium accuracy is between 3 Å and 5.5 Å, and low accuracy is above 5.5 Å. The score suggests that the peptide of MJE1 falls within a medium (75%–80%) accurate. This suggests that the small peptide of MJE1 is definitely significant to cause an adequate structural or conformational changes resulting to a change in effect or function of a receptor. A peptide with moderate affinity may still trigger strong downstream effects if the system amplifies the signal. Also, if the peptide is present at high concentration, it could cause a significant interaction with the receptor. Furthermore, some proteins/receptors only need transient interactions to be activated. We observed that the interaction with the active peptide‐protein surface could yield significant changes. This discovery suggests that the small peptides of *MJE1* have the potential to be investigated therapeutically.

The objective of our study did not include the quantification of the interaction in vivo. Instead, we focused on the identification of active peptide and the protein residues that showed significant interaction. In this study, we were able to demonstrate that the formation of an interaction between the *MJE1* and *CFH* receptor could be targeted therapeutically. Also, the potential identified in this study is strengthen by that fact that *MJE1* has drug‐like ability as reported in the ADME results. This finding necessitates further investigation in mouse, zebrafish or non‐human primate model. By being able to transform this peptide to a compound that can be administered intravenously of subcutaneously, in the future, we could investigate its therapeutic effects targeted against ageing‐related diseases, particularly delivered to the tissue/organ where *CFH* is most expressed.

The process of identifying the *CFH* from the Bgee data is supported in literature by many researchers. After annotating an RNA‐seq data, researchers could address the data base on specific objective they wish to achieve. RNA‐seq can reveal novel, or previously unannotated transcripts. Our primary objective was to narrow down genes by translational value; we reasoned that therapeutic targets genes should have been shared among the older donors while absent in the younger donors. We achieved the identification of CFH based on the objective and the filtering criteria outlined in the method section. TPM is a more accurate statistic when calculating gene expression comparisons across samples.

The *CFH* gene encodes the protein for CFH. It is a 155‐kDa glycoprotein containing 20 complement control protein modules, also known as short consensus repeats (SCRs) [32]. Factor H is a soluble protein composed of multiple related globular domains, which share about 60% of the amino acid identity and contain about 60 amino acids in each domain [32]. Factor H modules 1–4 have a specific role in complement regulation, working to recognise common epitopes exposed on glycosaminoglycans and glycosaminoglycan–protein complexes [33]. This function enables Factor H to identify host cells and anchor itself to their surface, which can control the alternative pathway while protecting the host [34]. Modules 19–20 are not involved in the regulatory function or the recognition of host patterns but serve as a stabilisation tool between the two regions. Modules 6–10 (commonly called the C‐terminal region) work closely with the N‐terminus in the regulatory mechanisms of factor H, enabling binding on C‐reactive proteins that have been opsonised by factor H [32]. The human complement regulator factor H regulates the immune response to disease and several inherited and acquired human diseases are promoted in part or in whole by defects in this gene of protein. Cytokines such as interferon‐gamma, IL‐6 and TNF‐*α* at normal or increased concentrations can stimulate the expression of factor H. At the same time, multiple dysregulations of factor H have been observed under pathological conditions [34]. Furthermore, the CFH system activation is restricted to areas of pathogen invasion or damaged cells, where it functions to promote phagocytosis and inflammation. The dysregulation of CFH activation is known to promote the development of a wide range of immune complex‐mediated pathologic conditions. These conditions encompass a diverse set of diseases ranging from haemolytic disorders and severe renal diseases to age‐related macular degeneration and Alzheimer’s disease. Of note, one of the earliest studies by Klein et al. reiterates the genetic role of CFH in age‐related macular degeneration (AMD). The study confirmed associations of the Y402H *CFH* gene variant with AMD in a population with a strong statistically significant frequency of *CFH* variant carriers having AMD [35]. A study confirmed that the human donor retinas graded for the presence and severity of AMD and genotyped for 10 common AMD risk variants in *CFH* and measured in the macular cells the relationship of the extent of damage compared to the genotype [36].

The Haung et al.’s paper comprehensively described the known mechanisms of ageing and ageing‐related diseases to date [37]. This encompasses genomic instability, epigenetic alterations, compromise of autophagy, mitochondrial dysfunction, cellular senescence, stem cell exhaustion, altered intercellular communication and deregulated nutrient sensing [37]. Of which, most have been linked to inflammation and oxidative stress. Taken together, the CFH was differentially expressed in the ageing group compared to the young donors. This data contributes to the fact that inflammation can enact ageing. Observing the interaction between the *MJE1* and CFH has shed light to the studies that investigated any of the plant derivatives containing *MJE1*, and targeting its responses against inflammation and immune responses cascade. By targeting the interaction of peptides with receptors is a key point affecting the efficacy of targeted drugs. Like the CFH, other receptors listed in Table 1 also have the potentials to be investigated and could reveal interesting findings that can improve our understanding of ageing processes.

Finally, our study is not without a limitation. We based our findings on computational algorithmic investigation. While this approach has tremendously been accurate and ushered in many advanced innovations in scientific research to date, we should highlight that genetic, epigenetic, ethnicity, age and environmental factors can significantly make an analysis and findings not applicable in vivo, unless being tailored to adapt to the conditions that affect the applicability. Because, in the actual sense, we are dealing with complex proteins that are highly prone to modifications and redundancy. Hence, an *MJE1-CFH* drug therapy will need to be investigated further and considering many other factors.

## 5. Conclusion

The findings from our study suggest plausible interactions and mechanisms that can be explored whilst the anti‐ageing potentials of *MJE1*. Our study highlighted the active peptides that can be modified in the design of novel candidate drugs that can target *CFH* activities in biological ageing related to immune responses. This information provides the foundation for advanced and cutting‐edge research in ageing.

## Ethics Statement

There is no ethical approval required for the study. Data were analysed from publicly available data and utilised appropriately.

## Conflicts of Interest

The authors declare no conflicts of interest.

## Author Contributions

Oluwafemi G. Oluwole conceptualised the study, organised data, performed analyses and drafted the manuscript. Ngalla Jillani, Afolake Arowolo and Solomon Umukoro equally provided expert contributions and editing of manuscript.

## Funding

No funding was received for this manuscript.

## Supporting Information

Additional supporting information can be found online in the Supporting Information section.

## Supporting information


**Supporting Information 1** For Figure S1, we used the NCBI BLAST. BLAST finds region of similarity between biological sequences. The program compares nucleotide or protein sequences to sequence databases and calculates the statistical significance. BLAST is a registered trademark of NCBI and described by Altschul SF, Gish W, Miller W, Myers EW, Lipman DJ, “Basic local alignment search tool”. Journal of Molecular Biology. 215(3): 403–410 (1990). Figure S1: The NCBI blastp multiple sequence alignment show a protein similarity score and plant species that produce similar proteins. The data also confirmed regions of sequence conservation across the species.

**Supporting Information 2** Figure S2 described an overview of the fastqc results. FastQC is a quality control tool for high throughput sequence data, providing a quick overview of potential problems in raw sequence data. Described by Andrews, S. (2010) FastQC: A Quality Control Tool for High Throughput Sequence Data. https://www.bioinformatics.babraham.ac.uk/projects/fastqc/. Figure S2: The quality control analysis results of the FASTQ files used for generating the differential gene expression data. After trimming and removal of duplicate, the selected bases had a Q > 20 score of 95.0% and a Q > 30 score of 97.6%.

**Supporting Information 3** Described by many foundational statisticians, we used the Q‐Q plot represented in Figure S3 to compare observed data’s quantiles to a theoretical distribution, for assessing the goodness‐of‐fit and distributional assumptions. Figure S3: The Q‐Q plot of normal quantile distribution of the expression values of all genes representing the small margin of variations.

## Data Availability

The data that support the findings of this study are available in EMBL’s European Bioinformatics Institute at https://www.ebi.ac.uk, reference number E‐MTAB‐2449. These data were derived from the following resources available in the public domain: Array expression, https://www.ebi.ac.uk/biostudies/arrayexpress/studies/E-MTAB.
