# Supplementary figures and images for: A Computational Investigation of Small Peptide of Methyl Jasmonate and Human Complement Factor in Ageing
Source: Int J Genomics. 2025 Oct 30;2025:9783996. doi: 10.1155/ijog/9783996 (PMC12575269; doi:10.1155/ijog/9783996)

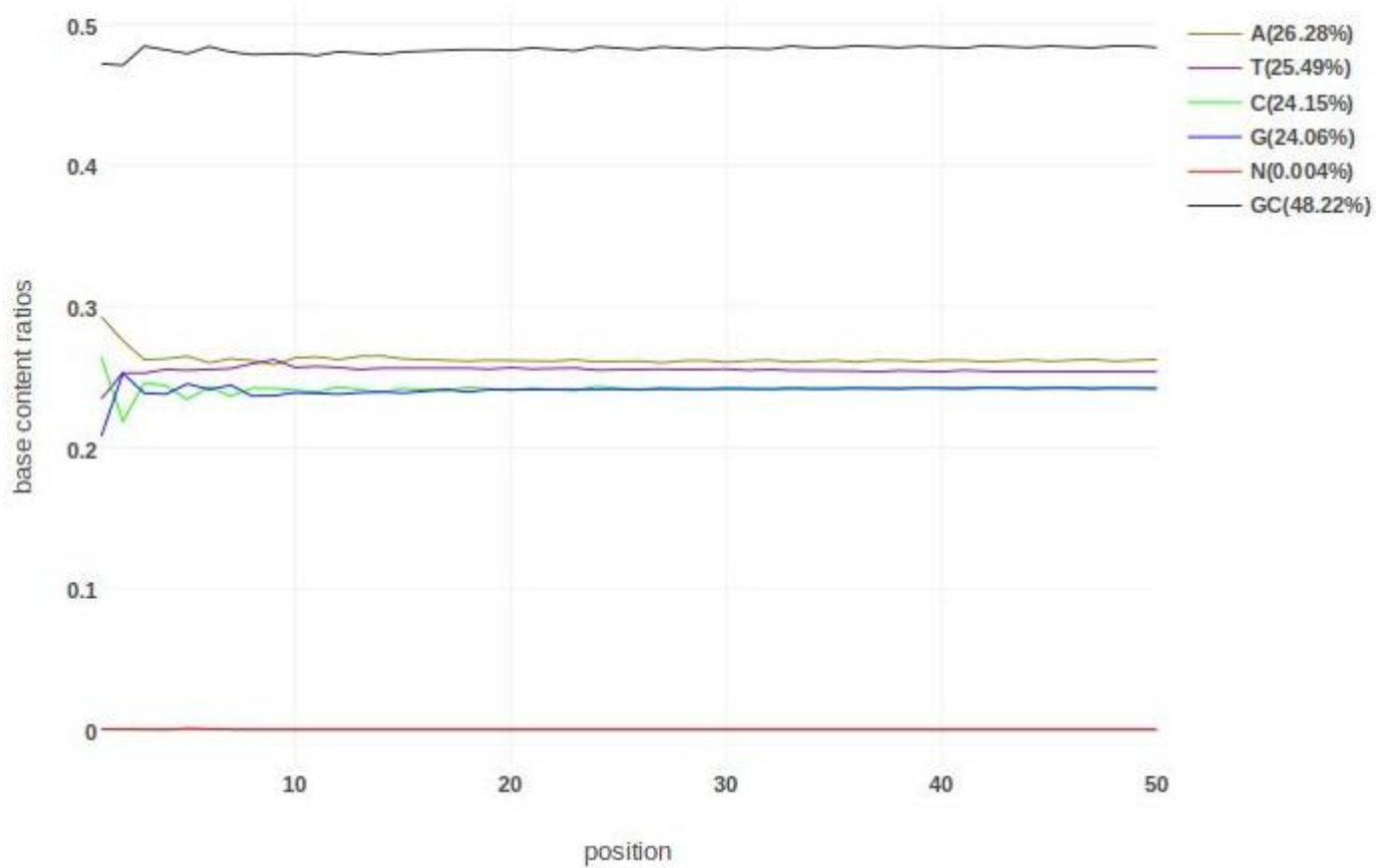

Supplement: Supplementary file 2 — Supporting Information 2 Figure S2 described an overview of the fastqc results. FastQC is a quality control tool for high throughput sequence data, providing a quick overview of potential problems in raw sequence data. Described by Andrews, S. (2010) FastQC: A Quality Control Tool for High Throughput Sequence Data. https://www.bioinformatics.babraham.ac.uk/projects/fastqc/. Figure S2: The quality control analysis results of the FASTQ files used for generating the differential gene expression data. After trimming and removal of duplicate, the selected bases had a Q > 20 score of 95.0% and a Q > 30 score of 97.6%. [file IJOG-2025-9783996-s003.pdf]

QQ plot

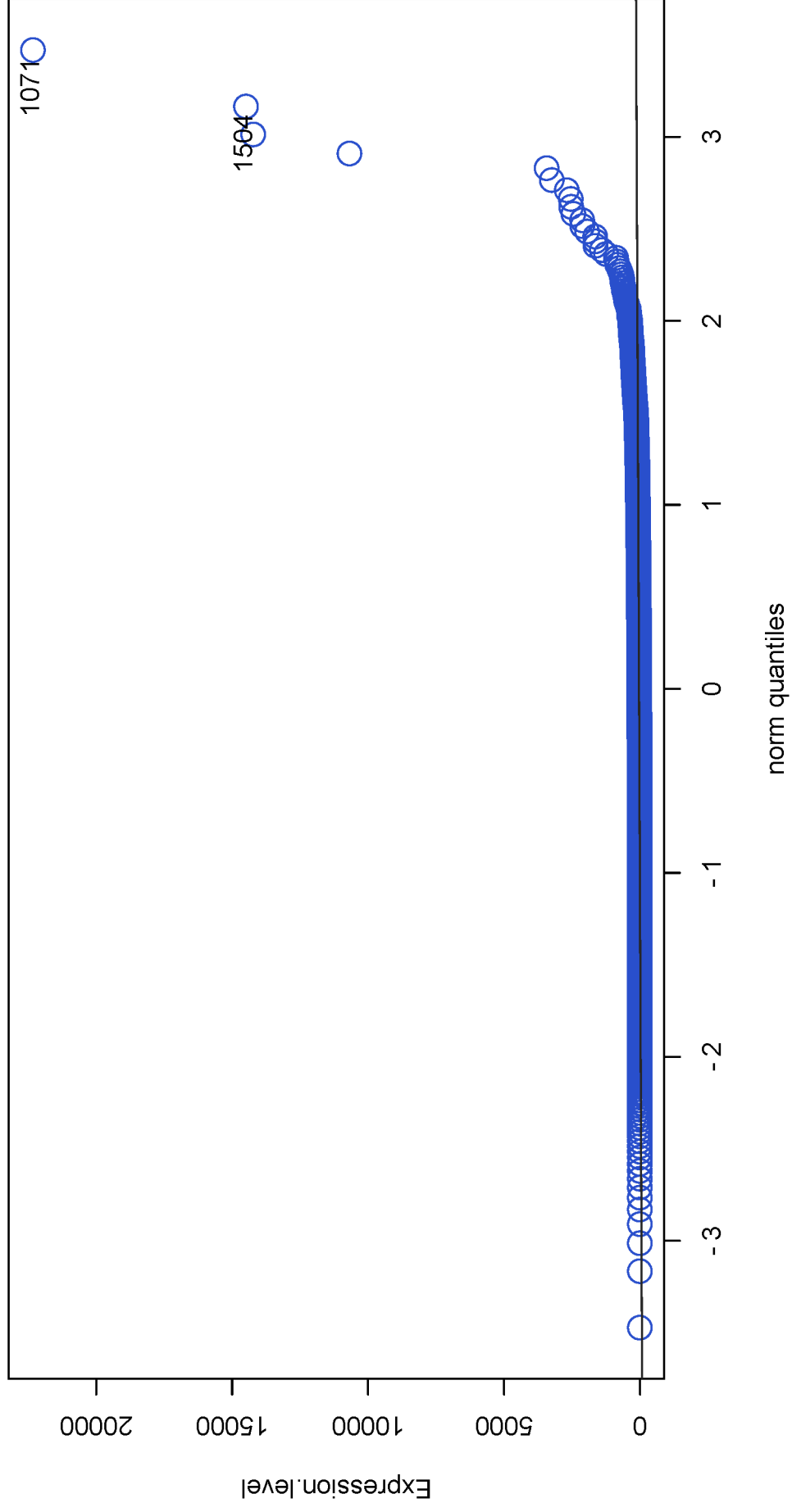

Supplement: Supplementary file 3 — Supporting Information 3 Described by many foundational statisticians, we used the Q‐Q plot represented in Figure S3 to compare observed data’s quantiles to a theoretical distribution, for assessing the goodness‐of‐fit and distributional assumptions. Figure S3: The Q‐Q plot of normal quantile distribution of the expression values of all genes representing the small margin of variations. [file IJOG-2025-9783996-s002.pdf]
